# The Role of B Cell Targeting in Chronic Graft-Versus-Host Disease

**DOI:** 10.3390/biomedicines5040061

**Published:** 2017-10-17

**Authors:** Ruben Rhoades, Sameh Gaballa

**Affiliations:** Department of Medical Oncology, Sidney Kimmel Cancer Center, Thomas Jefferson University, Philadelphia, PA 19107, USA; ruben.rhoades@jefferson.edu

**Keywords:** chronic graft-versus-host disease, B cell, rituximab, ibrutinib

## Abstract

Chronic graft-versus-host disease (cGVHD) is a leading cause of late morbidity and mortality following allogeneic stem cell transplantation. Current therapies, including corticosteroids and calcineurin inhibitors, are only effective in roughly 50% of cases; therefore, new treatment strategies are under investigation. What was previously felt to be a T cell disease has more recently been shown to involve activation of both T and B cells, as well as a number of cytokines. With a better understanding of its pathophysiology have come more expansive preclinical and clinical trials, many focused on B cell signaling. This report briefly reviews our current understanding of cGVHD pathophysiology and reviews clinical and preclinical trials with B cell-targeted agents.

## 1. Introduction

Chronic graft-versus-host disease (cGVHD) continues to be a major cause of morbidity and mortality following allogeneic hematopoietic stem cell transplantation (SCT) [1]. The cumulative incidence ranges from 20–77% [2], but is rising as factors that increase the rate of cGVHD—such as older recipient age, use of donor peripheral blood, and non-HLA identical donors—become more common, and transplant-related mortality decreases [3,4]. Assessing the response to therapy and interpreting clinical trials is made difficult by the lack of standardized definitions and response criteria for cGVHD [5], however, it has been widely reported that cGVHD adversely impacts treatment-related mortality and overall survival following allogeneic SCT [6,7].

The pathophysiology of cGVHD is complex [3,8] and incompletely understood and as a result, effective therapies and controlled trials are lacking [9]. Long thought to be a T cell disease, emerging evidence supports a role for B cells in the development of cGVHD [10,11], which carries important implications for prevention and treatment. Below we will briefly review the pathophysiology of cGVHD with a focus on B cell mechanisms, then we will outline both preclinical and clinical trial data on B cell-targeted therapies for the prevention and treatment of cGVHD.

## 2. Pathogenesis of Chronic Graft-Versus-Host Disease (cGVHD)

The presentation of cGVHD shares similarities with other autoimmune disorders, including lichen planus, scleroderma, bronchiolitis obliterans (BO), primary biliary cirrhosis, immune cytopenias, and chronic immunodeficiency [1,10]. It commonly presents within one year of allogeneic SCT, with a median time of 4–4.5 months following transplantation [12]. The most common manifestations are in the skin, mouth, liver, eyes, lung, GI tract, joints, and hematopoietic system [3]. Historically, cGVHD was classified as limited versus extensive, however, given its limitations, a number of classification and grading systems were introduced, including the Johns Hopkins model, the CIBMTR grading system, and the NIH consensus criteria for GVHD sensitivity. The NIH criteria, which include “diagnostic features” requiring no further workup to diagnose cGVHD and “distinctive features” requiring tissue confirmation for diagnosis, are widely used to diagnose cGVHD [13].

Given its overlap with a number of distinct autoimmune disorders, it follows that cGVHD is a complex, heterogeneous disease. While a major predictor of cGVHD is the development of prior acute GVHD (aGVHD), the pathogenesis of cGVHD involves more than simply prolongation of aGVHD [2]. aGVHD is primarily a T cell disease, occurring as a result of donor T cell activation in response to major or minor histocompatibility mismatch or gene polymorphisms. Donor-derived T cells are activated primarily through Th1 cytokines (IL-2, IFN-γ, and TNF-α) and then migrate from lymphoid tissue to target organs, where they cause damage to epithelial cells via apoptosis and cytokine release [14]. There is a direct relationship between the acute and chronic forms of GVHD. Approximately two-thirds of patients undergoing allogeneic SCT who develop cGVHD had earlier aGVHD [15,16], which suggests a prominent role for T cells in cGVHD pathogenesis. Proposed mechanisms include: late manifestations of alloreactive donor T cells; thymic damage during aGVHD causing T cell dysregulation and failure to delete autoreactive T cells [8]; a downstream effect of immunosuppressive treatment of aGVHD; or an associated but independent epiphenomenon [16]. Th17 cells and their primary cytokine, IL-17, have been implicated in sclerodermatous cGVHD [17] and high levels of IL-17 have been found in skin cGVHD [10]. Unlike in aGVHD, however, Th2 cytokines seem to predominate in cGVHD [10]. Furthermore, one third of patients develop cGVHD without any prior history of aGVHD. More recent research suggests cGVHD involves a complex interplay between T and B cells.

### B Cell Role in cGVHD Pathogenesis

Animal and human studies have in recent years demonstrated a prominent role for B cells in cGVHD development. An early suggestion of this came from the one study demonstrating autoantibodies in patients with cGVHD [18]. In another study of 121 male patients receiving an allogeneic SCT from female donors, antibodies directed against minor histocompatibility antigens encoded by genes on the Y chromosome were found in 52% of recipients, and these correlated with cGVHD [19]. A relationship between T and B cells was demonstrated in murine cGVHD models by Zhang et al., who showed that development of cGVHD in mice required both donor CD4^+^ CD25^−^ T cells and B cells [20]. Preclinical studies have shown that germinal centers (GC), where T follicular helper (TFH) cells interact with B cells, are increased in size and number in mice with cGVHD, and that development of cGVHD requires GC formation [21,22,23]. However, it remains unclear if murine models of cGVHD can accurately represent the pathophysiology of cGVHD in humans receiving allogeneic SCT. B cell homeostasis has also been implicated in cGVHD development. B cell activating factor (BAFF) plays an important role in B cell tolerance by limiting B cell receptor (BCR)-mediated apoptosis [24]. A study of BCR transgenic mice showed that BAFF overexpression selectively promoted survival of autoreactive B cells by rescuing them from peripheral deletion [25]. High levels of BAFF after allogeneic SCT, coupled with depletion of B cells by the conditioning regimen, prevent apoptosis of polyreactive B cells and may play a role in the development of cGVHD [24]. Additionally, lymphopenia leads to higher BAFF concentrations because naïve B cells are unavailable to bind soluble BAFF [24], while restoration of the naïve B cell component seems to be protective of cGVHD [26]. A study of cGVHD patients by Sarantopoulis et al. further supports the role of altered B cell homeostasis in cGVHD development [26]. The authors studied 20 patients with cGVHD who were previously treated with the anti-CD20 monoclonal antibody rituximab, 11 of whom had stable or improved cGVHD following treatment and nine of whom had progressive cGVHD. They found that all patients developed the expected profound B lymphopenia after rituximab therapy, but that the group with progressive cGVHD continued to have B cell depletion after a median of 25 months, while the responders recovered their peripheral B cell pools, which comprised primarily naïve IgD^+^ B cells. Similarly, BAFF levels increased in all patients following rituximab, but decreased in the responsive patients while remaining persistently elevated in the unresponsive group. Responders to rituximab had significant decreases in CD27^+^ B cells—which are antigen-experienced memory B cells—whereas the B cells of unresponsive patients demonstrated an activated phenotype [26]. Taken together, these findings suggest that skewing of the BAFF/B cell ratio and survival of activated autoreactive B cells promote cGVHD development. These results were confirmed in a larger prospective study of post-SCT rituximab prophylaxis, in which patients who went on to develop cGVHD had significantly lower B cell numbers and a higher BAFF/B cell ratio compared to those who did not develop cGVHD [27]. These studies, along with data suggesting that higher BAFF levels predict subsequent development of cGVHD [28], make BAFF a potentially useful biomarker for development of cGVHD, as well as a possible target for treating it.

Enhanced B cell activation and signaling has also been implicated in cGVHD. CD27^+^ B cells in cGVHD have been shown to constitutively produce IgG, which is distinct from healthy controls, in whom antigen or BCR stimulation is required to secrete antibody [24]. Allen et al. found that pre-GC CD27^+^ B cells in patients with active cGVHD were significantly larger than CD27^−^ cells, suggesting activation [29]. A later study also showed that B cells in cGVHD are hyper-responsive in response to antigen stimulation, and that the BCR signaling components spleen tyrosine kinase (Syk) and B cell linker protein (BLNK) were elevated at baseline. Further, they found that after initiation of BCR signaling, phosphorylation by Syk and BLNK were elevated compared to patients without cGVHD [30].

Regulatory B cells (Bregs), a subset of B cells capable of attenuating the immune response under inflammatory conditions, may possibly play a role in cGVHD development [31]. Bregs exert their inhibitory effect via multiple mechanisms, including secretion of the regulatory cytokines IL-10 and TGF-β and direct cell-to-cell interaction with pathogenic T cells [31]. IL-10 producing B cells have been shown to be deficient in human cGVHD patients [32]. Sarvaria et al. studied Bregs in cord blood, as well as in patients with and without cGVHD [33]. They found that Bregs were enriched in cord blood and were upregulated following allogeneic cord blood transplantation, but that Bregs were deficient in patients who developed cGVHD.

## 3. B Cell Targeting in the Treatment and Prevention of cGVHD

For decades, the mainstays of therapy for cGVHD have been meticulous organ-specific care and corticosteroids, often for at least one year in moderate-severe cGVHD (1). Prolonged corticosteroid therapy exposes patients to significant side effects, therefore, the calcineurin inhibitors cyclosporine and tacrolimus are often added. Still, 50–60% of patients have recurrent cGVHD symptoms within two years and require second-line therapy [5]. Many immunosuppressive agents have been studied in combination with corticosteroids—including mycophenolate mofetil, azathioprine, thalidomide, and hydroxychloroquine—with no benefit [1]. No current standard exists for second-line, steroid-refractory cGVHD [11].

### 3.1. B Cell Depletion in the Treatment and Prevention of cGVHD

#### 3.1.1. Rituximab

In an effort to improve upon these primarily T cell-directed therapies, other approaches have targeted B cells. The efficacy of B cell targeted agents in cGVHD was discovered somewhat incidentally. Ratanatharathorn et al. used rituximab in a patient with refractory thrombocytopenia and oral and ocular manifestations of cGVHD following allogeneic SCT, and found that, in addition to an improvement in her platelet count, her cGVHD symptoms also abated and immunosuppression was successfully stopped [34]. Since then, multiple small case series have demonstrated mixed results in steroid-refractory cGVHD [35,36,37,38,39,40,41,42].

In order to better understand the role of rituximab in treating cGVHD, multiple prospective studies have evaluated rituximab in refractory cGVHD, the largest of which by Cutler et al. included 20 patients treated with rituximab (375 mg/m^2^) for between one and three cycles, with a 70% response rate and two CRs at one year of follow-up. The median dose of prednisone was reduced from 40 mg/day to 10 mg/day, and the authors showed a sustained decrease in antibody titers to minor histocompatibility antigens encoded on the Y chromosome [43]. A small prospective study with seven patients demonstrated a slightly lower overall response rate (ORR) of 43% [44]. Another prospective study of 20 patients with cGVHD who received one four-week course of rituximab showed an ORR of 62%, with increased B cell number in responders [45]. More recently, a prospective phase II trial using either imatinib or rituximab for sclerodermatous cGVHD demonstrated a response in 19/72 (26%) of patients. High levels of CD27^+^ activated B cells before treatment predicted response in patients receiving rituximab, but not those receiving imatinib [46].

Rituximab has also been studied in the first-line setting in combination with corticosteroids. A study of 35 treatment-naïve patients with cGVHD showed a clinically meaningful response rate—defined as a PR or CR with prednisone <0.25 mg/kg/day—of 43%, which was sustained through one year [47]. Predictors of response included high levels of pre-treatment naïve CD19^+^ CD27^−^ B cells and low BAFF levels. IgG and IgM antibodies and specific B cells to male-specific H-Y antigens were measured in the 15 male recipients from female donors, and among patients who were positive for H-Y IgG and IgM before rituximab, all responders had undetectable levels following treatment. Meanwhile, three of four non-responders remained positive for H-Y IgG. A similar trend was observed in H-Y antigen specific B cells [47].

Last, rituximab has been evaluated as a preventive strategy for the development of cGVHD. The largest phase II trial carried out to date included 65 patients who received rituximab infusions at three, six, nine, and 12 months post-allogeneic SCT [27]. At two years post-transplantation, the rates of cGVHD and steroid-requiring cGVHD were 48% and 31%, respectively, both of which were lower than a control cohort. The rates decreased further to 35% and 23%, respectively, in patients with a sibling donor. Further, this study included only patients receiving peripheral blood SCT, who have a greater risk of cGVHD development. Treatment-related mortality (5% vs. 19%) and overall survival (71% vs. 56%) at four years were both significantly better in the rituximab-receiving group than the control cohort. The authors also noted that the BAFF/B cell ratio was significantly higher in patients who developed cGVHD (27), suggesting that rituximab exerts its protective effect partly through preserving B cell homeostasis. Another prospective study of rituximab given weekly from days +56–77 post-transplantation reported a cGVHD incidence of 20% [48], though these patients all received conditioning with antithymocyte globulin (ATG), which is associated with lower rates of cGVHD [49]. In this study, rituximab was associated with total prevention of H-Y antibodies in the 10 male recipients from female donors [49]. Not all studies of rituximab prophylaxis have demonstrated an effect on cGVHD development. A phase II trial in 84 patients demonstrated no effect of rituximab on cGVHD development at one year (46%, vs. 42% in the group not receiving rituximab). This study was marked by very high rates of aGVHD requiring an amendment of the protocol that mandated the use of ATG, making interpretation of the results more difficult [50]. Finally, a large retrospective CIBMTR study comparing rituximab-containing conditioning regimens to regimens without rituximab in B-NHL patients showed no effect on cGVHD [51]. The mixed evidence with rituximab may be in part because of the deleterious effect on B cell homeostasis by inducing profound B lymphopenia.

#### 3.1.2. Ofatumumab and Bortezomib

A phase I trial of the humanized anti-CD20 monoclonal antibody ofatumumab in 12 cGVHD patients with a new prednisone requirement showed a promising ORR of 67% (one CR, seven PR, one stable disease). A sustained decrease in prednisone dose was also observed at three and six months of follow-up [52]. A phase II study is ongoing.

The proteasome inhibitor bortezomib has been evaluated as a strategy to prevent and treat cGVHD. It functions as an immunomodulator which can reduce alloreactive T cells and antigen-presenting cells [53], but also depletes plasma cells and can inhibit the B cell response [54]. Koreth et al. first demonstrated that bortezomib may help reduce cGVHD associated with HLA-mismatched unrelated donor SCT [53]. In this phase I/II trial, a bortezomib dose of 1.3 mg/m^2^ was first selected based on maximum tolerated dose, and then given on Days +1, +4, and +7. In comparison to a contemporaneous cohort of patients receiving grafts from matched unrelated donors, the study patients had similar rates of cGVHD, non-relapse mortality, progression-free survival, and overall survival at one year [46]. The same authors then undertook a prospective phase II trial of 1st line bortezomib in the treatment of cGVHD. The study included 20 evaluable patients who received three cycles of bortezomib (on days 1, 8, 15, and 22 of five-week cycles), among whom the ORR at 15 weeks was 80% (two CR, 14 PR). Prednisone dosing was significantly reduced by week 15 [55].

Pai et al. have studied bortezomib in preclinical murine models, and are undertaking a single arm phase I trial in patients with refractory or severe cGVHD [54]. In an early analysis of 10 patients, 6/10 received at least 60% of planned bortezomib doses, and of those patients all but one achieved at least a partial remission. The corticosteroid dose was also reduced successfully [54].

### 3.2. B Cell Signaling Inhibition in the Treatment and Prophylaxis of cGVHD

#### 3.2.1. Imatinib

The Bcr-Abl tyrosine kinase inhibitors imatinib, dasatinib, and nilotinib—used primarily in the treatment of chronic myelogenous leukemia and Philadelphia chromosome-positive acute lymphoblastic leukemia (Ph+ ALL)—inhibit BCR signaling and B cell proliferation via inhibition of multiple tyrosine kinases, and may also dampen B cell immune responses via inhibition of Bruton’s tyrosine kinase (BTK), a key enzyme in B cell signaling, proliferation, and cell survival [56,57]. These properties have generated interest in this class of drugs for the prevention and treatment of cGVHD. A phase I/II trial of imatinib in 19 patients with treatment-refractory cGVHD with fibrotic features showed an ORR of 79%, though eight patients had to discontinue the drug and impact on steroid use was not measured [58]. A larger phase II study with 39 patients showed a response rate of 46%, defined as PR or minor responses (MR) with steroid sparing [59]. Response to therapy correlated with three-year overall survival. A phase II crossover trial of either rituximab or imatinib showed similar efficacy between the two agents in cutaneous sclerodermatous cGVHD, with a clinical response rate to imatinib of 26% [46]. Dasatinib has also been reported in case series to be effective in patients with cGVHD refractory to imatinib [60].

#### 3.2.2. Ibrutinib

The BTK inhibitor ibrutinib, which has been approved in CLL, mantle cell lymphoma, and Waldenstrom’s macroglobulinemia, has been studied in cGVHD for its potent B cell signaling inhibition properties. In a preclinical murine allogeneic SCT model, ibrutinib was shown to limit development of sclerodermatous cGVHD and to improve existing skin lesions. It had a similar protective and therapeutic effect on pulmonary BO (61). In vivo studies showed that ibrutinib blocked both T and B cell activation and that it limited the GC reaction and immunoglobulin deposition in the lungs. Ibrutinib also acts as an IL-2 inducible T cell kinase (ITK) inhibitor, and this study confirmed that both BTK and ITK are required for the development of cGVHD [61].

In response to preclinical findings, ibrutinib was studied in a phase II trial [62]. Miklos et al. found that in 42 patients with steroid-refractory cGVHD (median prior regimens = 2), ibrutinib demonstrated an ORR of 67%, with a durable response at ≥20 weeks and ≥32 weeks of 71% and 48%, respectively. Five of the 28 responders successfully discontinued corticosteroids entirely. Both patient-reported symptom score and markers of inflammation improved in patients treated with ibrutinib [62]. Based on the results of this trial, ibrutinib received FDA approval in August 2017 for second-line treatment of cGVHD.

### 3.3. Future Directions

#### 3.3.1. Syk Inhibition

Syk is a downstream BCR signaling enzyme that promotes B cell proliferation and survival. Syk is overexpressed at baseline in cGVHD patients, and demonstrates greater phosphorylation following BCR signaling compared to patients without cGVHD [30]. Fostamitinib, a potent small molecule inhibitor of Syk, is being studied in a preclinical murine model with pulmonary cGVHD and has been found to restore normal lung function in these mice [23]. Further, it decreased the number of splenic GC B cells and GC reactions, which are crucial for cGVHD development [21]. In another preclinical study, fostamitinib did not have an effect on murine sclerodermatous cGVHD, but did induce death by apoptosis in human cGVHD cells [63]. Clinical trials using fostamitinib (NCT02611063) and the Syk inhibitor entospletinib (NCT02701634) are ongoing in the treatment of cGVHD.

#### 3.3.2. IL-21/IL-21R Inhibition

IL-21 is a cytokine involved in cell proliferation with receptors on T, B, and natural killer cells. Monoclonal antibodies against IL-21 and its receptor—as well as inducible T cell costimulatory (ICOS) and CD40, which are necessary for GC formation—have demonstrated a reduction in GC formation and BO development in mice [22].

#### 3.3.3. Rho-Associated Kinase 2 (ROCK2) Inhibition

Rho-associated kinase 2 (ROCK2) is a member of the ROCK family of serine-threonine kinases. In addition to regulating cell movement, ROCK2 was recently found to regulate IL-21 and IL-17 secretion, making it an enticing target in cGVHD. A selective ROCK2 inhibitor, KD025, has been studied in murine models of full multisystem cGVHD with BO (major histocompatibility mismatch) and more limited sclerodermatous cGVHD (minor histocompatibility mismatch). Mice treated with KD025 had a normalization of lung function and reduced antibody deposition in the lungs. Further, spleens of treated mice showed reduced TFH cells and an increase in Treg cells, as well as a reduction of STAT3 phosphorylation [64].

#### 3.3.4. BCL6 Inhibition

BCL6 is a transcription factor that promotes GC development and TFH cell differentiation. Murine studies suggest an increase in BCL6+ splenic T cells in cGVHD, and the small molecule BCL6 inhibitor 79-6 may reverse BO and decrease GC B cells [23].

#### 3.3.5. EZH2 Inhibition

EZH2 is a lysine methyltransferase that works in concert with BCL6 to promote GC development. It methylates histone and leads to gene repression, and is upregulated during the GC reaction, allowing proliferation and affinity maturation in activated B cells [65]. Sarantopoulos et al. demonstrated that deleting EZH2 from early B cells or splenic T cells prevents cGVHD in mice, and then showed that the selective EZH2 inhibitor JQ-E can reverse lung cGVHD and fibrosis [23].

## 4. Conclusions

cGVHD is a complex process involving an interplay between T and B cells, as well as other cytokines and immune cells. Current, first-line therapy with corticosteroids with or without calcineurin inhibitors is often ineffective, therefore, ongoing investigation in preclinical and clinical trials is required to better understand the disease and improve its treatment. More recent discoveries have highlighted a prominent role for B cells in the development of cGVHD, prompting the study of B cell-targeted therapies for the prevention and treatment of cGVHD. Rituximab has shown mixed efficacy in steroid-refractory cGVHD and may help prevent its development. Understanding of B cell signaling has led to the study and recent FDA approval of the BTK inhibitor ibrutinib in refractory cGVHD. Looking ahead, more preclinical studies will be needed to identify novel agents aimed at B cell proliferation and GC development, and promising drugs such as the Syk inhibitors fostamitinib and entospletinib require ongoing study in clinical trials as first- and second-line therapies.

## References

[1] Flowers M.E.D., Martin P.J. (2015). How we treat chronic graft-versus-host disease. Blood.

[2] Perez-Simon J.A., Sanchez-Abarca I., Diez-Campelo M., Caballero D., San Miguel J. (2006). Chronic Graft-Versus-Host Disease. Drugs.

[3] Lee S.J., Vogelsang G., Flowers M.E.D. (2003). Chronic Graft-versus-Host Disease. Biol. Blood Marrow Transplant..

[4] Mohty M., Kuentz M., Michallet M., Bourhis J.-H., Sutton L., Jouet J.-P., Attal M., Bordigoni P., Cahn J.-Y., Blaise D. (2002). Chronic graft-versus-host disease after allogeneic blood stem cell transplantation: Long-term results of a randomized study. Blood.

[5] Inamoto Y., Flowers M.E.D., Sandmaier B.M., Aki S.Z., Carpenter P.A., Lee S.J., Sotrer B.E., Martin P.J. (2014). Failure-free survival after initial systemic treatment of chronic graft-versus-host disease. Blood.

[6] Boyiadzis M., Arora M., Klein J.P., Hassebroek A., Hemmer M., Urbano-Ispizua A., Antin J.H., Bolwell B.J., Cahn J.-Y., Cairo M.S. (2015). Impact of Graft-versus-Host Disease on Late Relapse and Survival on 7,489 Patients after Myeloablative Allogeneic Hematopoietic Cell Transplantation for Leukemia. Clin. Cancer Res..

[7] Martin P.J., Counts G.W., Appelbaum F.R., Lee S.J., Sanders J.E., Degg H.J., Flowers M.E.D., Syrjala K.L., Hansen J.A., Storb R.F. (2010). Life Expectancy in Patients Surviving More Than 5 Years After Hematopoietic Cell Transplantation. J. Clin. Oncol..

[8] Bhushan V., Collins R.H. (2003). Chronic Graft-vs-Host Disease. J. Am. Med. Assoc..

[9] Martin P.J., Lee S.J., Przepiorka D., Horowitz M.M., Koreth J., Vogelsang G.B., Walker I., Carpenter P.A., Griffith L.M., Akpek G. (2015). National Institutes of Health Consensus Development Project on Criteria for Clinical Trials in Chronic Graft-versus-Host Disease: VI. The 2014 Clinical Trial Design Working Group Report. Biol. Blood Marrow Transplant..

[10] MacDonald K.P.A., Hill G.R., Blazar B.R. (2017). Chronic graft-versus-host disease: Biological insights from preclinical and clinical studies. Blood.

[11] Cutler C.S., Koreth J., Ritz J. (2017). Mechanistic approaches for the prevention and treatment of chronic GVHD. Blood.

[12] Lee S.J., Klein J.P., Barrett A.J., Ringden O., Antin J.H., Cahn J.-Y., Carabasi M.H., Gale R.P., Giralt S., Hale G.A. (2002). Severity of chronic graft-versus-host disease: Association with treatment-related mortality and relapse. Blood.

[13] Filipovic A.H., Weisdorf D., Pavletic S., Socie G., Wingard J.R., Lee S.J., Martin P., Chien J., Przepiorka D., Couriel D. (2005). National Institutes of Health Consensus Development Project on Criteria for Clinical Trials in Chronic Graft-versus-Host Disease: I. Diagnosis and Staging Working Group Report. Biol. Blood Marrow Transplant..

[14] Sun Y., Tawara I., Toubai T., Reddy P. (2007). Pathophysiology of Acute Graft-vs-Host Disease: Recent Advances. Transl. Res..

[15] Ozawa S., Nakaseko C., Nishimura M., Maruta A., Cho R., Ohwada C., Sakamaki H., Sao H., Mori S., Okamoto S. (2007). Chronic graft-versus-host disease after allogeneic bone marrow transplantation from an unrelated donor: Incidence, risk factors and association with relapse. A report from the Japan Marrow Donor Program. Br. J. Haematol..

[16] Lee S.J. (2005). New approaches for preventing and treating chronic graft-versus-host disease. Blood.

[17] Hill G.R., Olver S.D., Kuns R.D., Varelias A., Raffelt N.C., Don A.L., Markey K.A., Wilson Y.A., Smyth M.J., Iwakura Y. (2010). Stem cell mobilization with G-CSF induces type 17 differentiation and promotes scleroderma. Blood.

[18] Kier P., Penner E., Bakos S., Kalhs P., Lechner K., Volc-Platzer B., Wesierska-Gadek J., Sauermann G., Gadner H., Emminger-Schmidmeier W. (1990). Autoantibodies in chronic GVHD: High prevalence of antinucleolar antibodies. Bone Marrow Transplant..

[19] Miklos D.B., Kim H.T., Miller K.H., Guo L., Zorn E., Lee S.J., Hochberg E.P., Wu C.J., Alyea E.P., Cutler C. (2005). Antibody responses to H-Y minor histocompatibility antigens correlate with chronic graft-versus-host disease and disease remission. Blood.

[20] Zhang C., Todorov I., Zhang Z., Liu Y., Kandeel F., Forman S., Strober S., Zeng D. (2006). Donor CD4+ T and B cells in transplants induce chronic graft-versus-host disease with autoimmune manifestations. Blood.

[21] Srinivasan M., Flynn R., Price A., Ranger A., Browning J.L., Taylor P.A., Ritz J., Antin J.H., Murphy W.J., Luznik L. (2012). Donor B-cell alloantibody deposition and germinal center formation are required for the development of murine chronic GVHD and bronchiolitis obliterans. Blood.

[22] Flynn R., Du J., Veenstra R.G., Reichenbach D.K., Panoskaltsis-Mortari A., Taylor P.A., Freeman G.J., Serody J.S., Murphy W.J., Munn D.H. (2014). Increased T follicular helper cells and germinal center B cells are required for cGVHD and bronchiolitis obliterans. Blood.

[23] Sarantopoulos S., Blazar B.R., Cutler C., Ritz J. (2015). B Cells in Chronic Graft versus Host Disease. Biol. Blood Marrow Transplant..

[24] Sarantopoulos S., Stevenson K.E., Kim H.T., Cutler C.S., Bhuiya N.S., Schowalter M., Ho V.T., Alyea E.P., Koreth J., Blazar B.R. (2009). Altered B-cell homeostasis and excess BAFF in human chronic graft-versus-host disease. Blood.

[25] Thien M., Phan T.G., Gardam S., Amesbury M., Basten A., Mackay F., Brink R. (2004). Excess BAFF rescues self-reactive B cells from peripheral deletion and allows them to enter forbidden follicular and marginal zone niches. Immunity.

[26] Sarantopoulis S., Stevenson K.E., Kim H.T., Washel W.S., Bhuiya N.S., Cutler C.S., Alyea E.P., Ho V.T., Soiffer R.J., Antin J.H. (2011). Recovery of B cell homeostasis after rituximab in chronic graft-versus-host disease. Blood.

[27] Cutler C., Kim H.T., Bindra B., Sarantopoulos S., Ho V.T., Chen Y.-B., Rosenblatt J., McDonough S., Watanaboonyongcharoen P., Armand P. (2013). Rituximab prophylaxis prevents corticosteroid-requiring chronic GVHD after allogeneic peripheral blood stem cell transplantation: Results of a phase 2 trial. Blood.

[28] Sarantopoulos S., Stevenson K.E., Kim H.T., Bhuiya N.S., Cutler C.S., Soiffer R.J., Antin J.H., Ritz J. (2007). High Levels of B-Cell Activating Factor in Patients With Active Chronic Graft-Versus-Host Disease. Clin. Cancer Res..

[29] Allen J.L., Fore M.S., Wooten J., Roehrs P.A., Bhuiya N.S., Hoffert T., Sharf A., Deal A.M., Armistead P., Coghill J. (2012). B cells from patients with chronic GVHD are activated and primed for survival via BAFF-mediated pathways. Blood.

[30] Allen J.L., Tata P.V., Fore M.S., Wooten J., Rudra S., Deal A.M., Sharf A., Hoffert T., Roehrs P.A., Shea T.C. (2014). Increased BCR responsiveness in B cells from patients with chronic GVHD. Blood.

[31] Mizoguchi A., Bhan A.K. (2006). A Case for Regulatory B Cells. J. Immunol..

[32] Khoder A., Sarvaria A., Alsuliman A., Chew C., Sekine T., Cooper N., Mielke S., de Lavallade H., Muftuoglu M., Curbelo I.F. (2014). Regulatory B cells are enriched within the IgM memory and transitional subsets in healthy donors but are deficient in chronic GVHD. Blood.

[33] Sarvaria A., Basar R., Mehta R.S., Shaim H., Muftuoglu M., Khoder A., Sekine T., Gokdemir E., Kondo K., Marin D. (2016). IL-10^+^ regulatory B cells are enriched in cord blood and may protect against cGVHD after cord blood transplantation. Blood.

[34] Ratanatharathorn V., Carson E., Reynolds C., Ayash L.J., Levine J., Yanik G., Silver S.M., Ferrara J.L.M., Uberti J.P. (2000). Anti-CD20 chimeric monoclonal antibody treatment of refractory immune-mediated thrombocytopenia in a patient with chronic graft-versus-host disease. Ann. Int. Med..

[35] Ratanatharathorn V., Ayash L., Reynolds C., Silver S., Reddy P., Becker M., Ferrara J.L., Uberti J.P. (2003). Treatment of chronic graft-versus-host disease with anti-CD20 chimeric monoclonal antibody. Biol. Blood Marrow Transplant..

[36] Canninga-van Dijk M.R., van der Straaten H.M., Fijnheer R., Sanders C.J., van den Tweel J.G., Verdonck L.F. (2004). Anti-CD20 monoclonal antibody treatment in 6 patients with therapy-refractory chronic graft-versus-host disease. Blood.

[37] Okamoto M., Okano A., Akamatsu S., Ashihara Inaba T., Takenaka H., Katoh N., Kishimoto S., Shimazaki C. (2006). Rituximab is effective for steroid-refractory sclerodermatous chronic graft-versus-host disease. Leukemia.

[38] Carella A.M., Biasco S., Nati S., Congiu A., Lerma E. (2007). Rituximab is effective for extensive steroid refractory chronic graft-vs-host-disease. Leuk. Lymphoma.

[39] Zaja F., Bacigalupo A., Patriarca F., Stanzani M., Van Lint M.T., Fili C., Scime R., Milone G., Flada M., Vener C. (2007). Treatment of refractory chronic GVHD with rituximab: A GITMO study. Bone Marrow Transplant..

[40] Mohty M., Marchetti N., El-Cheikh J., Faucher C., Furst S., Blaise D. (2008). Rituximab as salvage therapy for refractory chronic GVHD. Bone Marrow Transplant..

[41] Von Bonin M., Oelschlagel U., Radke J., Stewart M., Ehninger G., Bornhauser M., Platzbecker U. (2008). Treatment of chronic steroid-refractory graft-versus-host disease with low-dose rituximab. Transplantation.

[42] Clavert A., Chevallier P., Guillaume T., Delauney J., Le Gouill S., Mahe B., Dubruille V., Gastinne T., Blin N., Moreau P. (2012). Safety and efficacy of rituximab in steroid-refractory chronic GVHD. Bone Marrow Transplant..

[43] Cutler C., Miklos D., Kim H.T., Tresiter N., Woo S.-B., Bienfang D., Klickstein L.B., Levin J., Miller K., Reynolds C. (2006). Rituximab for steroid-refractory chronic graft-versus-host disease. Blood.

[44] Teshima T., Nagafuji K., Henzan H., Miyamura K., Takase K., Hidaka M., Miyamoto T., Takenaka K., Akashi K., Harada M. (2009). Rituximab for the treatment of corticosteroid-refractory chronic graft-versus-host disease. Int. J. Hematol..

[45] Van Dorp S., Resemann H.K., te Boome L.C.J., Lokhorst H.M., Petersen E.J., Minnema M.C., Ebeling S.B., de Weger R.A., van Dijk M.R., Meijer E. (2011). Rituximab treatment in chronic GVHD: Clinical efficacy associates with restoring a physiological B-cell balance. Biol. Blood Marrow Transplant..

[46] Arai S., Pidala J., Pusic I., Chai X., Jaglowski S., Khera N., Palmer J., Chen G.L., Jagasia M., Mayer S.A. (2016). A Randomized Phase II Crossover Study of Imatinib or Rituximab for Cutaneous Sclerosis after Hematopoietic Cell Transplantation. Clin. Cancer Res..

[47] Sahaf B., Arai S., Otani J., Schoenrock K., Logan A., Miklos D.B. (2013). Rituximab provides steroid-sparing therapy in new-onset chronic graft-versus-host disease. Biol. Blood Marrow Transplant..

[48] Arai S., Sahaf B., Narasimhan B., Chen G.L., Jones C.D., Lowsky R., Shizuru J.A., Johnston L.J., Laport G.G., Weng W.-K. (2012). Prophylactic rituximab after allogeneic transplantation decreased B-cell alloimmunity with low chronic GVHD incidence. Blood.

[49] Nakasone H., Sahaf B., Miklos D.B. (2015). Therapeutic benefits targeting B-cells in chronic graft-versus-host disease. Inter. J. Hematol..

[50] Glass B., Hasenkamp J., Wulf G., Dreger P., Pfreundschuh M., Gramatzki M., Silling G., Wilhelm C., Zeis M., Gorlitz A. (2014). Rituximab after lymphoma-directed conditioning and allogeneic stem-cell transplantation for relapsed and refractory aggressive non-Hodgkin lymphoma (DSHNHL R3): An open-label, randomised, phase 2 trial. Lancet Oncol..

[51] Epperla N., Ahn K.W., Ahmed S., Jagasia M., DiGilio A., Devine S.M., Jaglowski S., Kennedy V., Rezvani A.R., Smith S.M. (2017). Rituximab-containing reduced-intensity conditioning improves progression-free survival following allogeneic transplantation in B cell non-Hodgkin lymphoma. J. Hematol. Oncol..

[52] Pidala J., Kim J., Betts B.C., Alsina M., Ayala E., Fernandez H.F., Field T., Kharfan-Dabaja M.A., Locke F.L., Mishra A. (2015). Ofatumumab in Combination with Glucocorticoids for Primary Therapy of Chronic Graft-versus-Host Disease: Phase I Trial Results. Biol. Blood Marrow Transplant..

[53] Koreth J., Stevenson K.E., Kim H.T., McDonough S.M., Bindra B., Armand P., Ho T., Cutler C., Blazar B.R., Antin J.H. (2012). Bortezomib-Based Graft-Versus-Host Disease Prophylaxis in HLA-Mismatched Unrelated Donor Transplantation. J. Clin. Oncol..

[54] Pai C.C.S., Chen M., Mirsoian A., Grossenbacher S.K., Tellez J., Ames E., Sun K., Jagdeo J., Blazar B.R., Murphy W.J. (2014). Treatment of chronic graft-versus-host disease with bortezomib. Blood.

[55] Herrera A.F., Kim H.T., Bindra B., Jones K.T., Alyea E.P., Armand P., Cutler C.S., Ho V.T., Nikiforow S., Blazar B.R. (2014). A Phase II Study of Bortezomib Plus Prednisone for Initial Therapy of Chronic Graft-versus-Host Disease. Biol. Blood Marrow Transplant..

[56] Olivieri J., Coluzzi S., Attolico I., Olivieri A. (2011). Tirosin kinase inhibitors in chronic graft versus host disease: from bench to bedside. Sci. World J..

[57] De Lavallade H., Khoder A., Hart M., Sarvaria A., Sekine T., Alsuliman A., Mielke S., Bazeos A., Stringaris K., Ali S. (2013). Tyrosine kinase inhibitors impair B-cell immune responses in CML through off-target inhibition of kinases important for cell signaling. Blood.

[58] Olivieri A., Locatelli F., Zecca M., Sanna A., Cimminiello M., Raimond R., Gini G., Mordini N., Balduzzi A., Leoni P. (2009). Imatinib for refractory chronic graft-versus-host disease with fibrotic features. Blood.

[59] Olivieri A., Cimminiello M., Corradini P., Mordini N., Fedele R., Selleri C., Onida F., Patriarca F., Pavone E., Svegliati S. (2013). Long-term outcome and prospective validation of NIH response criteria in 39 patients receiving imatinib for steroid-refractory chronic GVHD. Blood.

[60] Sánchez-Ortega I., Servitje O., Arnan M., Orti G., Peralta T., Manresa F., Duarte R.F. (2012). Dasatinib as salvage therapy for steroid refractory and imatinib resistant or intolerant sclerotic chronic graft-versus-host disease. Biol. Blood Marrow Transplant..

[61] Dubovsky J., Flynn R., Du J., Harrington B.K., Zhong Y., Kaffenberger B., Yang C., Towns W.H., Lehman A., Johnson A.J. (2014). Ibrutinib treatment ameliorates murine chronic graft-versus-host disease. J. Clin. Investig..

[62] Miklos D., Cutler C.S., Arora M., Waller E.K., Jagasia M., Pusic I., Flowers M.E., Logan A.C., Nakamura R., Blazar B.R. Multicenter Open-Label Phase 2 Study of Ibrutinib in Chronic Graft Versus Host Disease (cGVHD) After Failure of Corticosteroids. Proceedings of the ASH 58th Annual Meeting.

[63] Flynn R., Allen J.L., Luznik L., MacDonald K.P., Paz K., Alexander K.A., Vulic A., Du J., Panoskaltsis-Mortari A., Taylor P.A. (2015). Targeting Syk-activated B cells in murine and human chronic graft-versus-host disease. Blood.

[64] Flynn R., Paz K., Du J., Reichenbach D.K., Taylor P.A., Panoskaltsis-Mortari A., Vulic A., Luznik L., MacDonald K.K., Hill G.R. (2016). Targeted Rho-associated kinase 2 inhibition suppresses murine and human chronic GVHD through a Stat3-dependent mechanism. Blood.

[65] Velichutina I., Shaknovich R., Geng H., Johnson N.A., Gascoyne R.D., Melnick A.M., Elemento O. (2010). EZH2-mediated epigenetic silencing in germinal center B cells contributes to proliferation and lymphomagenesis. Blood.

